# Physiological characteristics of blood pressure responses after combined exercise in elderly hypertensive patients: a systematic review and meta-analysis

**DOI:** 10.3389/fcvm.2024.1404127

**Published:** 2024-10-25

**Authors:** Zhiheng Li, Moran LV, Zhen Li, Wei Gao, Ming Li

**Affiliations:** School of Physical Education and Sport Science, Fujian Normal University, Fuzhou, China

**Keywords:** combined exercise, aerobic exercise, resistance exercise, essential hypertension, elderly patients

## Abstract

**Objective:**

The aim of this investigation is to explore the efficacy of combined exercise in elderly patients with hypertension. Moreover, we aim to delve into the underlying mechanisms governing blood pressure regulation, with the objective of promoting the adoption of this exercise regimen among elderly hypertensive individuals.

**Methods:**

In our study, we conducted a thorough search across multiple databases, including PubMed, Web of Science, Cochrane Library, Embase, and Scopus. This extensive search resulted in the preliminary screening of 2,347 articles. Among these, 9 studies were carefully selected for an in-depth analysis. For our meta-analysis, we employed Review Manager 5.3 and Stata 15.0, enabling us to perform detailed subgroup analyses and assess the possibility of publication bias.

**Results:**

In comparison to the control group (*n* = 194), individuals enrolled in the combined exercise group (*n* = 200) exhibited a notable decrease in both resting systolic blood pressure (SBP) [weighted mean difference (WMD) = −11.17 mm Hg, 95% confidence interval (CI) (−17.13, −5.22), *Z* = 3.68, *P* < 0.05] and diastolic blood pressure (DBP) [WMD = −5.93 mm Hg, 95% CI (−9.24, −2.61), *Z* = 3.51, *P* < 0.05]. Nonetheless, no statistically significant alteration was observed in pulse pressure (PP) [WMD = −9.05 mm Hg, 95% CI (−22.65, 4.55), *Z* = 1.3, *P* = 0.192]. Further subgroup analyses elucidated that combined exercise regimens, characterized by aerobic training intensities below 85% of HRmax, durations of up to 12 weeks, weekly frequencies of either ≥3 or <3 sessions, total session times under 60 min, and a sequence of aerobic exercise followed by resistance training (AE-RT), were particularly effective in enhancing SBP and DBP among elderly patients with hypertension. Additionally, regular engagement in combined exercise led to significant improvements in SBP and DBP across individuals aged 60–70, those older than 70 years, and regardless of whether participants were using antihypertensive medications or not.

**Conclusion:**

Combined exercise serves as an efficacious adjunctive therapy for reducing blood pressure among elderly individuals with hypertension, exerting beneficial influences on multiple physiological mechanisms pertinent to blood pressure regulation. Moreover, the integration of aerobic exercise with resistance training presents a more varied training program, thereby eliciting wider-ranging positive effects on both the physical and mental well-being of elderly patients afflicted with hypertension.

## 1 Introduction

Hypertension is broadly acknowledged as a widespread chronic condition and is a significant concern for global health. As of January 2021, the worldwide prevalence of hypertension surpassed 1.28 billion individuals, it is estimated that the number of individuals with hypertension globally will rise to 1.5 billion by 2025 (1). Beyond its substantial impact on individual health, hypertension also contributes to a significant economic burden, affecting both national economies and family finances. Hypertension, defined as a clinical syndrome marked by elevated systemic arterial pressure, is categorized into two main types: primary and secondary. The cause of primary hypertension is not yet clear, but many studies suggest that it could be associated with environmental and genetic factors, accounting for about 90% of all hypertension cases (2). Concurrently, the increase in the global elderly population poses new challenges. This population has a broad age range and distinctly heterogeneous health conditions. The cumulative effect of aging and diseases presents numerous challenges to clinical diagnosis and chronic disease management (3). Aging of the heart and vessels enhance the risks of developing diseases such as hypertension, arteriosclerosis, atrial fibrillation, and heart failure (4). Patients diagnosed with grade-1 hypertension or prehypertension typically remain asymptomatic, despite consistently elevated blood vessel pressure. Over time, this sustained pressure exerts negative effects on blood vessel physiology, potentially compromising brain, heart, and kidney function (5). When patients are diagnosed with grade 2 hypertension or above, long-term treatment with antihypertensive drugs is required, along with close monitoring of blood pressure. For hypertensive patients who are untreated or inadequately treated, they are prone to developing various diseases, and their risk of mortality increases (6). Previously, practitioners in the medical field have used drugs to improve the problems caused by high blood pressure.

Exercise is an effective means of controlling blood pressure and marks the initiation of lifestyle modifications for hypertensive patients. It achieves blood pressure reduction by improving vascular and renal function (7, 8). From a physiological perspective, both aerobic and resistance exercises exert crucial roles in the mechanisms of blood pressure reduction, and they may even have synergistic effects, constituting common exercise prescriptions for hypertension (9). Relevant studies have shown that aerobic exercise is the most effective exercise intervention for decreasing blood pressure, with an average reduction of 6–10 mm Hg, whereas resistance exercise achieves a reduction of approximately 2–3 mm Hg (10). Aerobic exercise can lower blood pressure and enhance vascular health by strengthening the cardiovascular system, dilating blood vessels, reducing peripheral vascular resistance, and improving cardiopulmonary health, thus decreasing cardiac metabolic risk factors such as obesity, dyslipidemia, and abnormal blood glucose levels (7). Resistance exercise, a form of exercise where the body overcomes external resistance to increase muscle strength, mass, and bone density, primarily achieves blood pressure reduction through physiological mechanisms such as muscle strengthening, promoting vasodilation, and enhancing cardiac function (11). Taylor et al. (2016) (12) demonstrated that specific resistance exercise modalities can diminish sympathetic nervous system activity while augmenting parasympathetic tone, consequently modulating cardiac rate and arterial pressure regulation. Research has further indicated that resistance training confers a multitude of health benefits. Specifically, it assists in managing blood pressure, accelerates the metabolism of sugars, fats, and proteins, enhances oxygen uptake, and plays a pivotal role in safeguarding myocardial tissue. By inhibiting ventricular remodeling and decreasing the left ventricular mass index, resistance training effectively mitigates functional impairments to critical organs, including the heart, brain, and kidneys (13), thereby ensuring stable regulation of both systolic and diastolic blood pressure. Moreover, resistance training is recognized as an effective strategy for augmenting muscle strength, muscle mass, and bone density (14). Consequently, the integration of aerobic and resistance exercises, commonly termed as combined exercise, emerges as an optimal form of physical activity for promoting overall human health.

Currently, clinical trials investigating the impact of exercise on hypertensive elderly populations predominantly focus on either aerobic or resistance modalities, with a paucity of research exploring their conjunctive use. A meta-analysis underscores the capacity of combined exercise programs to regulate hypertension, revealing reductions in both systolic and diastolic blood pressure ranging from 0 to 4 mmHg (15). Our study not only synthesizes data from diverse investigations to confirm the effectiveness of combined aerobic and resistance exercises in older adults but also delves into the unique characteristics of these programs and the underlying mechanisms responsible for blood pressure reduction. This necessitates a thorough examination of evidence derived from multiple randomized controlled trials to rigorously and objectively evaluate the efficacy of the combined approach in managing hypertension among the elderly population. The outcomes of this study provide novel and valuable insights into the management of blood pressure and the enhancement of quality of life for older individuals.

## 2 Methods

### 2.1 Search strategy

This investigation was meticulously conducted in strict accordance with the Preferred Reporting Items for Systematic Reviews and Meta-Analyses (PRISMA) guidelines. Our adherence to these standards guarantees the utmost quality in reporting. Following the PICOS principle, the research question was divided into five components to guide the literature search. Both domestic and international literature published until August 2024 were searched in various databases. Search terms encompassed “Resistance Training”, “Training, Resistance”, "Hypertension”, “Aerobic training”, “High Intensity Interval Training”, “Physical Activity”, “Activities, Physical”, “Exercises”, “Combined exercise”, “elderly” and “Concurrent training”. Boolean operators (AND, OR, NOT) were utilized to construct search strategies. Thorough searches were performed in databases including PubMed, Cochrane Library, Embase, Web of Science, and Scopus to locate pertinent randomized controlled trials. Two independent researchers screened the literature using document management software, NoteExpress, with any disagreements resolved through consultation with a third researcher. Initially, duplicate studies were eliminated in the screening process, followed by the exclusion of clearly irrelevant studies based on the examination of titles and abstracts. Subsequently, full-text articles were evaluated to ascertain their suitability for inclusion in this study.

### 2.2 Research inclusion criteria

The selection criteria for studies included in this analysis were defined as follows: (1) participants aged 60 years or older; (2) adherence to a randomized controlled trial (RCT) format in study design; (3) implementation of a primary intervention in the experimental group comprising a regimen integrating aerobic and resistance exercises; (4) The outcome measures must include reports of at least one of the parameters: systolic blood pressure, diastolic blood pressure, or pulse pressure.

(5) Changes in blood pressure should be reported using auscultation, oscillometry, or ambulatory blood pressure monitoring, with blood pressure values reported both before and after the intervention.

### 2.3 Study exclusion criteria

Studies with the following characteristics were excluded: (1) nonoriginal research; (2) lack of outcome indicators; (3) incomplete articles that are reviews, reports, opinions or letters; and (4) documents not in Chinese or English.

### 2.4 Data extraction

Two researchers extracted data from the included literature, collected basic information of the experimental and control groups, and summarized the study design, training intensity, frequency, number of groups, intervention duration, baseline information and outcome indicators of the included literature.

### 2.5 Literature quality assessment

Two researchers independently conducted quality assessments of each study using the TESTEX (Tool for the Evaluation of Studies and Reporting Quality in Exercise) checklist (16). The assessment checklist is primarily divided into two sections: research quality (items 1–5) and research reporting quality (items 6–11). For each item on the TESTEX checklist, one point is awarded if the criterion is met; notably, item 6 contains three sub-items and item 8 contains two sub-items, with each sub-item also awarding one point if the criterion is met. Based on the total score, the studies are classified as low quality (0–5 points), moderate quality (6–10 points), and high quality (11–15 points).

### 2.6 Data loss processing

In some studies, relevant data were lacking. Emails were sent to the corresponding authors to request the missing data. If no response was received from the corresponding authors, those studies were excluded from this meta-analysis.

### 2.7 Statistical analysis

For this analysis, we established specific inclusion criteria for the selected studies. Continuous data from these studies were synthesized using meta-analytic techniques with RevMan 5.3 and Stata 15.0 software. Mean values and standard deviations (SD) were extracted to construct forest plots for analysis. Given that outcome measures were continuous variables reported in uniform units, we employed weighted mean difference (WMD) along with 95% confidence intervals (95% CI) for statistical evaluation. To evaluate the heterogeneity among different studies, heterogeneity tests (*I*^2^) were conducted. An *I*^2^ value greater than 50% indicates substantial heterogeneity between each study, necessitating further analyses such as subgroup analysis and meta-regression to investigate the sources of heterogeneity. Sensitivity analysis was performed to examine the robustness of the study. For the test of combined statistics, the probability value (*P*) of the statistic was derived based on the *Z*-value. When the *P*-value is less than 0.05, it suggests that the results from combining multiple studies are statistically significant. Egger's test was used to assess whether there was publication bias in this study.

## 3 Results

### 3.1 Literature screening process and results

**Figure 1:**
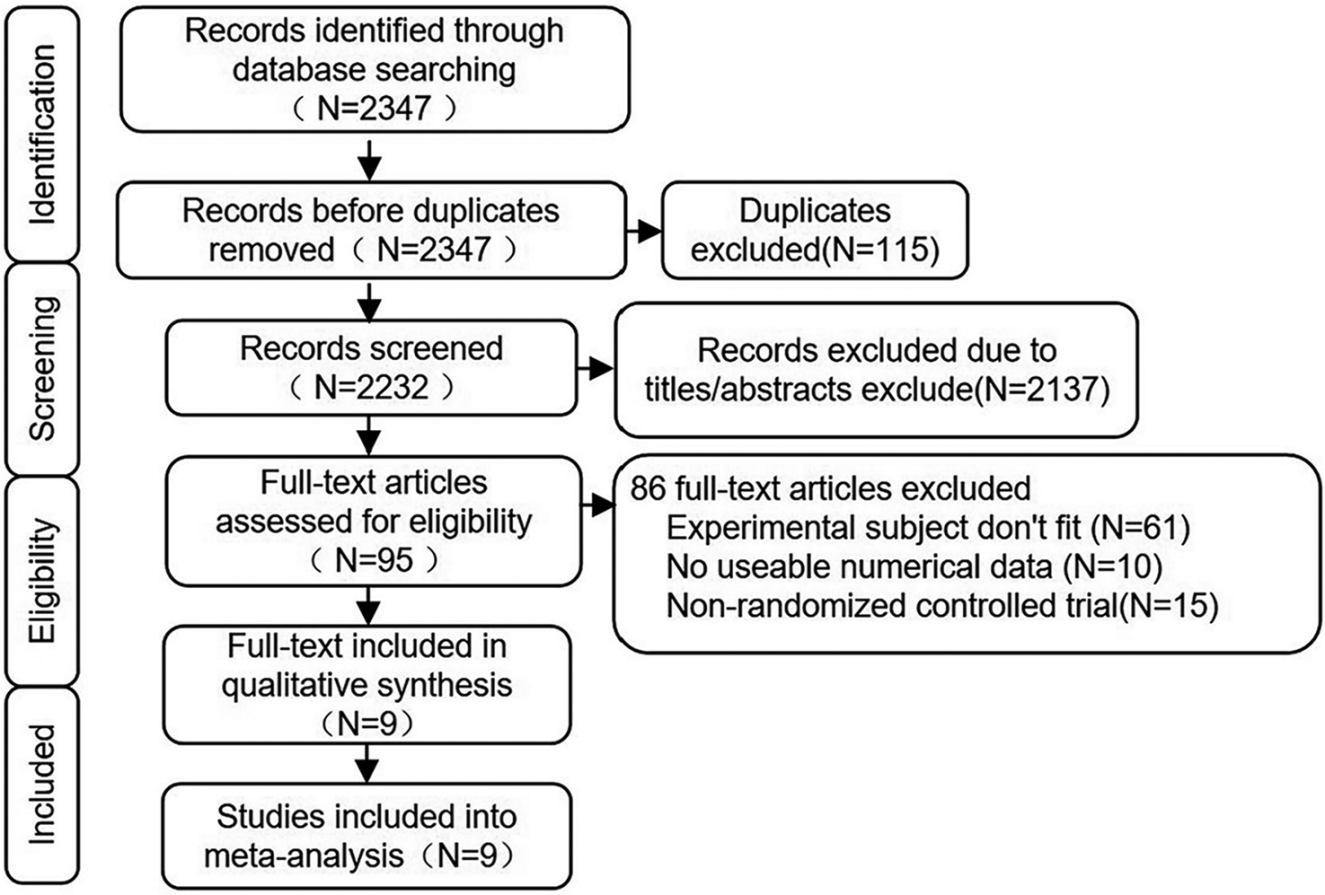
Steps of the literature search.

The literature screening process followed the PRISMA flowchart, and the retrieval process is illustrated in Figure 1. A total of 2,347 articles were retrieved from PubMed, Web of Science, Cochrane Library, Embase, Scopus, CNKI (China National Knowledge Internet), and Wanfang Database. Among these, 115 articles were duplicates. Based on the titles and abstracts, 2,137 articles were clearly identified as not meeting the requirements. After thoroughly reviewing the remaining 95 articles, 61 were found to be irrelevant to the theme of this study, 10 did not provide clear outcome measures for systolic or diastolic blood pressure, and 15 were not randomized controlled trials. Therefore, only 9 articles met the criteria for this study.

### 3.2 Research overview

This study included a total of 9 studies (17–25), involving 368 participants in the experimental and control groups (Table 1, Basic information of subjects). There were 200 participants in the combined exercise group and 194 in the control group, all of whom were individuals without professional sports experience or long-term exercise habits. As shown in Table 2, the intervention measures in all 9 studies were combined exercise. Among them, 5 studies used walking as the form of aerobic exercise, 3 studies indicated treadmill training as the aerobic exercise mode, and 1 study used cycling as the aerobic exercise method. Resistance exercises, as recorded in the studies, encompassed both upper and lower body movements, although the exact exercises varied across the individual studies. In each of the included studies, baseline measurements of basic health-related and anthropometric variables, cardiopulmonary function, resting blood pressure and other variables were collected before the start of the study, and participants underwent a familiarization protocol of the exercises several times after all initial assessments. In the formal intervention stage, the adherence rate was >85%. Subjects performed combined exercise two or three times a week, with 48 h separating each session.

**Table 1:** Basic information of subjects.

Study	Year	Number of participants	Type of hypertension	Mean age	Baseline SBP (mmHg)	Baseline DBP (mmHg)	Intake of antihypertensive medication
Barone et al.	2009	CEG: 16 CG: 14	Stage 1	64.6 ± 5.7	140 ± 8 142 ± 8	77 ± 7 76 ± 9	NO
Sousa et al.	2013	CEG: 13 CG: 10	Stage 1	69.1 ± 5	148.5 ± 15.1 138.8 ± 15.9	75.71 ± 7.52 76.17 ± 5.86	NO
EDUARDO S. dos Santos et al.	2014	CEG: 20 CG: 20	Stage 2	62.6 ± 2.5 63.1 ± 2.3	166.05 ± 8.06 160.70 ± 9.12	89.85 ± 4.82 95.03 ± 3.01	YES
Miura et al.	2015	CEG: 45 CG: 47	Stage 1	72.9 ± 5.7	150.0 ± 9.1 149.7 ± 9.0	83.5 ± 5.9 84.4 ± 6.9	NO
Son et al.,	2016	CEG: 10 CG: 10	Stage 1	74.7 ± 2	145.73 ± 2.98 147.62 ± 3.14	95.03 ± 3.01 97 ± 4.05	NO
Ratree Ruangthai et al. (1)	2019	CEG: 16 CG: 12	Stage 1	67 ± 5.8	142 ± 12.2 140.6 ± 18.2	81.7 ± 5.6 82.5 ± 10.1	YES
Ratree Ruangthai et al. (2)	2019	CEG: 16 CG: 12	Stage 1	66.7 ± 5.8	140.3 ± 18.53 142.06 ± 11.47	81.18 ± 6.18 82.06 ± 10.59	YES
de Oliveira et al.	2019	CEG: 16 CG: 13	Stage 1	62.65 ± 6.4	128.93 ± 8.43 130.65 ± 2.68	75.71 ± 7.52 76.17 ± 5.87	YES
Bruno et al.	2024	CEG: 13 CG: 13	Stage 1	70.25 ± 8.25	135.7 ± 14.3 129.1 ± 12.4	78.9 ± 8.4 79.7 ± 7.1	YES

Note: CEG, combined exercise group; CG, control group; SBP, systolic blood pressure; DBP, diastolic blood pressure.

**Table 2:** Intervention program of combined exercise.

Study	Total number of participants	Intervention duration (week)	AE/RT time (min)	Intensity of training	Training frequency	Type of training
Barone et al.	51	24 weeks	45/NR	AE: 60%–90% HRmax RT: 50% 1-RM	3 days/week	AE: walking RT: shoulder press, leg press, bench press, leg curls, leg extensions, and lat pull-downs.
Sousa et al.	16	32 weeks	30/NR	AE: 60%–80% HRR RT: 65%–75% 1-RM	3 days/week	AE: running RT: (1) bench press, leg press, lat pull-down, leg extension, military press, leg curl, arm curl. (2) Abdominal and erector spinae muscle groups targeted through floor exercises.
EDUARDO S. Dos Santos et al.	20	16 weeks	20/NR	AE: 65–75% HRR RT: 100%–120% 10-RM	3 days/week	AE: treadmill training RT: Barbell bench press, inclined leg press at 45 degrees, back extension, extension of the leg, bicep curl, ankle dorsiflexion, and side shoulder raise.
Miura et al.	45	12 weeks	20/40	AE: 44% HRR RT: NR	2 days/week	AE: bicycle training RT: Circuit training incorporating resistance bands or free weights; seated resistance training
Son et al.,	20	12 weeks	30/40	AE: 40%–70% HRR RT: RPE 11–16	3 days/week	AE: walking RT: Upper-body resistance band training: seated cable rows, hammer curls, anterior deltoid raises, concentric bicep curls, bench press variations; lower-body resistance band training: psoas major activation, gluteus maximus extension, gastrocnemius elevation, seated leg presses, barbell back squats.
Ruangthai (1)	16	12 weeks	20/20	AE: 50%–70% HRmax RT: 50–80% 1-RM	3 days/week	AE: walking RT: Barbell back squats, hanging leg raises, leg extension machine exercises, single-leg hamstring curls, hip adduction/abduction exercises, glute kickbacks, military press, flat bench barbell press, barbell bicep curls, bench triceps dips, side bends, abdominal crunches, and lumbar hyperextensions.
Ruangthai (2)	16	12 weeks	20/20	AE: 50%–70% HRmax RT: 50%–80% 1-RM	3 days/week	AE: walking RT: Barbell squats, elevated leg lifts, seated leg extensions, single-leg curls, thigh abduction/adduction exercises, posterior leg lifts, overhead press, flat bench press, dumbbell curls, tricep bench dips, oblique side bends, crunches, and prone back extensions.
de Oliveira et al.	13	8 weeks	25/25	AE: 70%–85% HRmax RT: RPE 5–7	3 days/week	AE: treadmill training RT: Upper extremities: cable rows, military press, bicep curls; lower extremities: leg extensions, hamstring curls, resistance band forward walks.
Bruno et al.	26	8 weeks	NR	AE: RPE 3 RT: RPE 3	2 days/week	AE: Walking RT: Vertical chest push, plantar flexion, sitting row, hip abduction, elbow flexion, torso flexion and chair squats

Note: AE, aerobic exercise; RT, resistance training; HRmax, maximum heart rate; HRR, heart rate reserve; 1-RM, one-repetition maximum; RPE, rating of perceiver exertion; MHR, maximum heart rate; HRR, heart rate reserve; NR, not reported.

### 3.3 Literature quality assessment and publication bias

As shown in Table 3, two researchers assessed the methodological quality of the 9 studies using the TESTEX checklist. Three studies (33.3%) were rated as “high quality,” five studies (55.6%) as “medium quality,” and one study (11.1%) as “low quality.” The common deduction items were as follows: (1) As indicated by questions 2 and 3, six studies (66.7%) did not report the method of random allocation or whether allocation concealment was used; (2) In question 5, six studies (66.7%) did not describe the use of blinding; (3) In question 6, five studies (55.6%) did not describe the attendance or completion rate of the participants; (4) In question 7, four studies (44.4%) did not perform intention-to-treat analysis for participants who withdrew; (5) In question 10, seven studies (77.8%) did not report whether exercise monitoring was conducted for the participants; (6) In question 11, eight studies (88.9%) did not regularly assess exercise intensity to ensure a constant relative exercise intensity. Egger's test was used to assess whether there was publication bias in this study, and no evidence of publication bias was found in the studies of SBP (*P* = 0.253) and DBP (*P* = 0.357). Egger's test cannot be conducted on PP due to the few included studies.

**Table 3:** Methodological quality assessment.

Study	Study quality					Study reporting							Overall	Judgement
	1	2	3	4	5	6	7	8	9	10	11	12		
Barone et al. (24)	1	0	0	1	0	0	1	2	1	0	0	1	7	Medium
Sousa et al. (19)	1	0	0	1	0	2	1	2	1	1	1	1	11	High
dos Santos et al. (20)	1	0	0	1	0	0	0	2	1	0	0	0	5	Low
Miura et al. (18)	1	0	0	1	0	0	0	2	1	0	0	1	6	Medium
Son et al. (21)	1	1	1	1	1	0	1	2	1	1	0	1	11	High
Ruangthai et al. (22) (1)	1	0	0	1	0	2	0	2	1	0	0	1	8	Medium
Ruangthai et al. (23) (2)	1	0	0	1	0	2	0	2	1	0	0	1	8	Medium
de Oliveira et al. (25)	1	1	1	1	1	0	1	2	1	0	0	1	10	Medium
Bavaresco Gambassi et al. (17)	1	1	1	1	1	1	1	2	1	0	0	1	11	High

### 3.4 Outcome measures

#### 3.4.1 Effect of combined exercise on SBP

**Figure 2:**
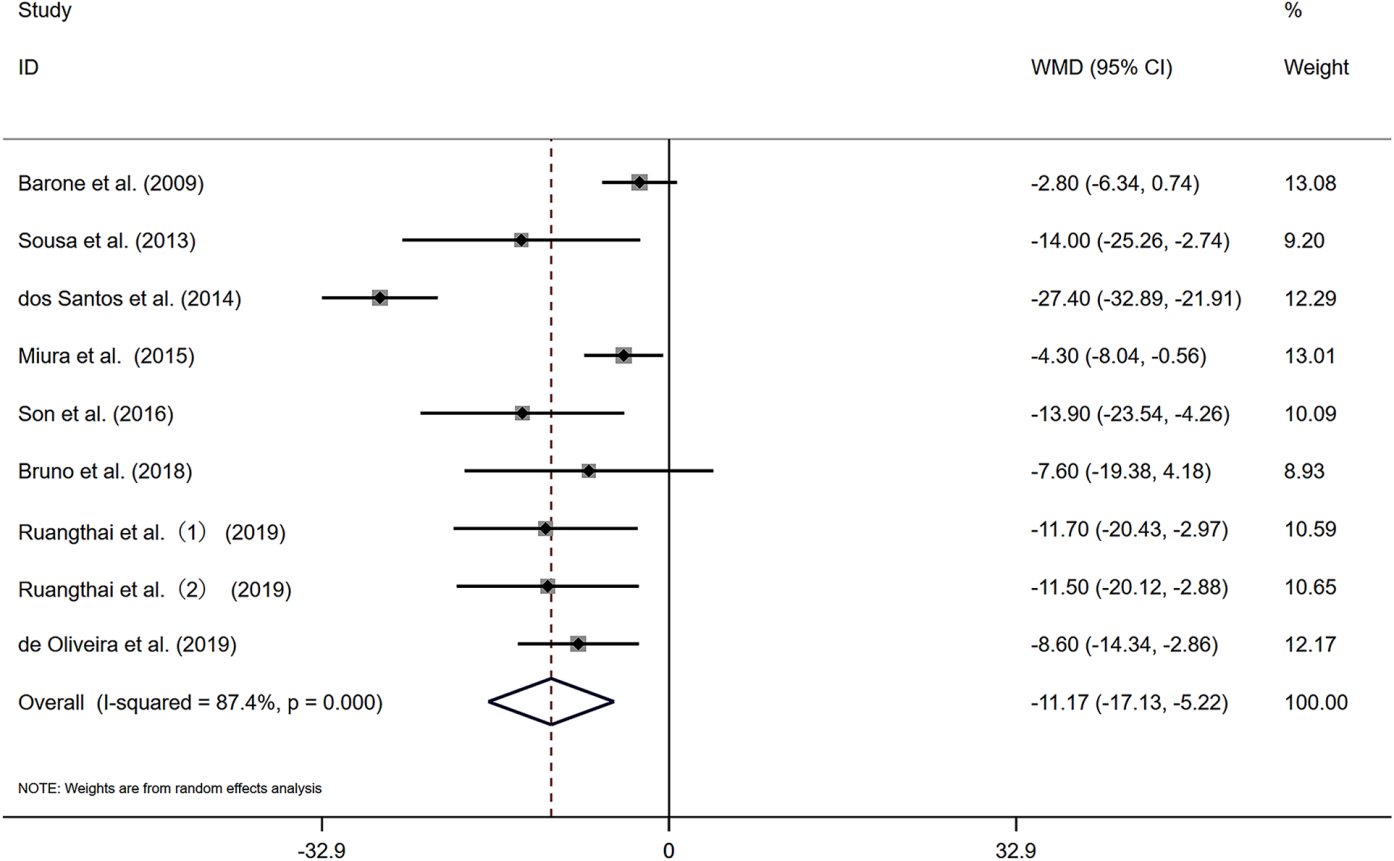
Forest plot of the effects of combined exercise on SBP.

Figure 2 presents nine studies that reported changes in resting SBP for 200 participants engaged in combined exercise interventions.The pooled results from the nine studies indicate that, compared with the control group, combined exercise had a positive effect on the SBP of the subjects [WMD = −11.17 mmHg, *I*^2^ = 87.4% (*P* < 0.05)]. Substantial heterogeneity was observed among the studies, and a random-effects model was applied. The pooled statistical results were statistically significant [*Z* = 3.68 (*P* < 0.05), 95% CI (−17.13, −5.22)], suggesting that combined exercise has a significant blood pressure-lowering effect on the SBP of elderly patients with hypertension. A sensitivity analysis (Supplementary Figure S1) revealed that the exclusion of any single study had minimal impact on the overall pooled effect size, indicating robustness of the findings.

#### 3.4.2 Effect of combined exercise on DBP

**Figure 3:**
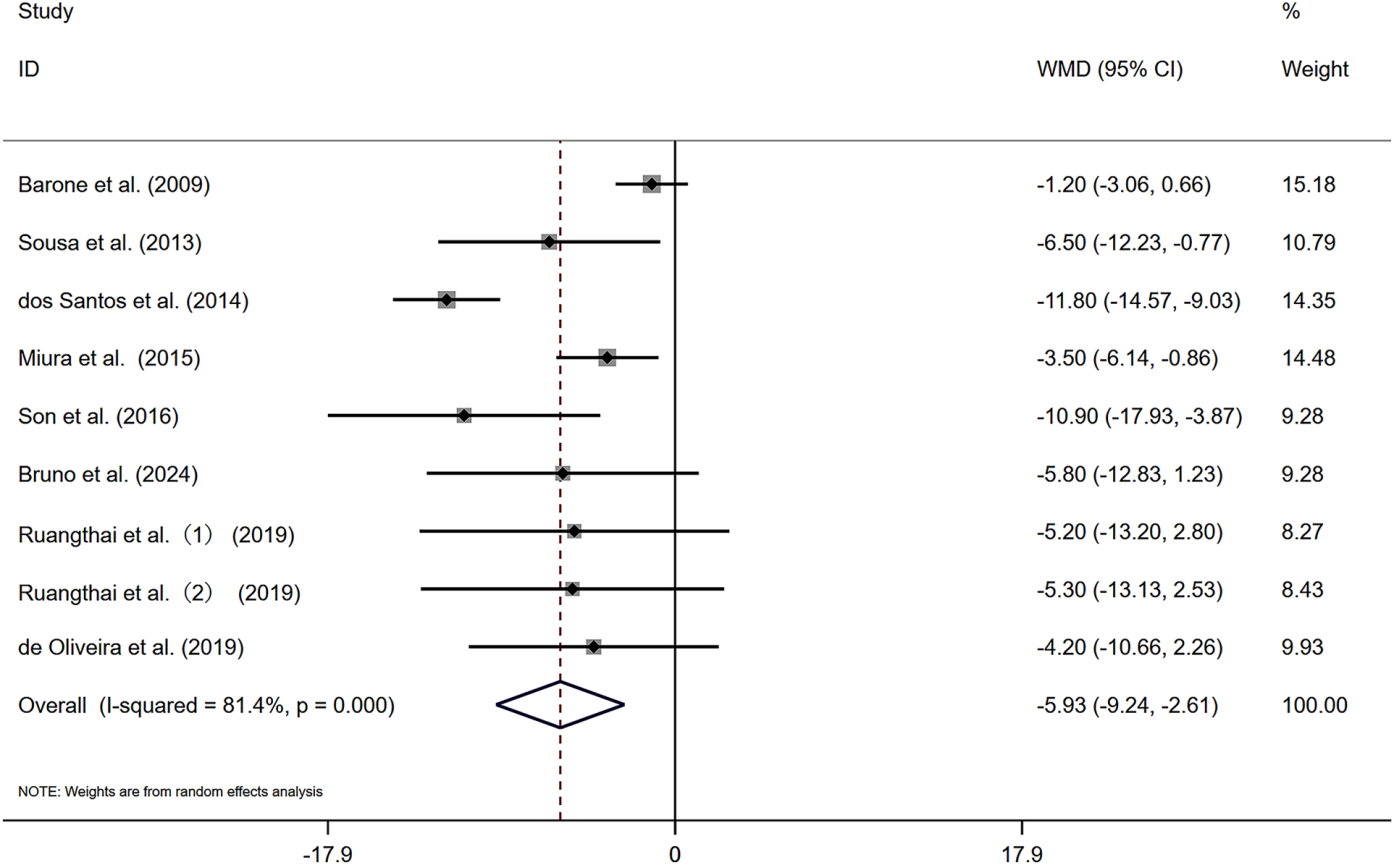
Forest plot of the effects of combined exercise on DBP.

Figure 3 illustrates that the nine included studies provided data on changes in resting diastolic blood pressure (DBP) among participants engaging in combined exercise, with a total of 200 individuals receiving the combined exercise intervention. The combined exercise had a positive effect on the DBP of the subjects [WMD = −5.93 mmHg, *I*^2^ = 81.4% (*P* < 0.05)]. Given the substantial heterogeneity observed among the studies, a random-effects model was employed. The pooled statistical results were statistically significant [*Z* = 3.51 (*P* < 0.05), 95% CI (−9.24, −2.61)], indicating that combined exercise effectively lowers the DBP of elderly patients with hypertension. A sensitivity analysis (Supplementary Figure S2) confirmed the robustness of these findings.

#### 3.4.3 Effect of combined exercise on PP

**Figure 4:**
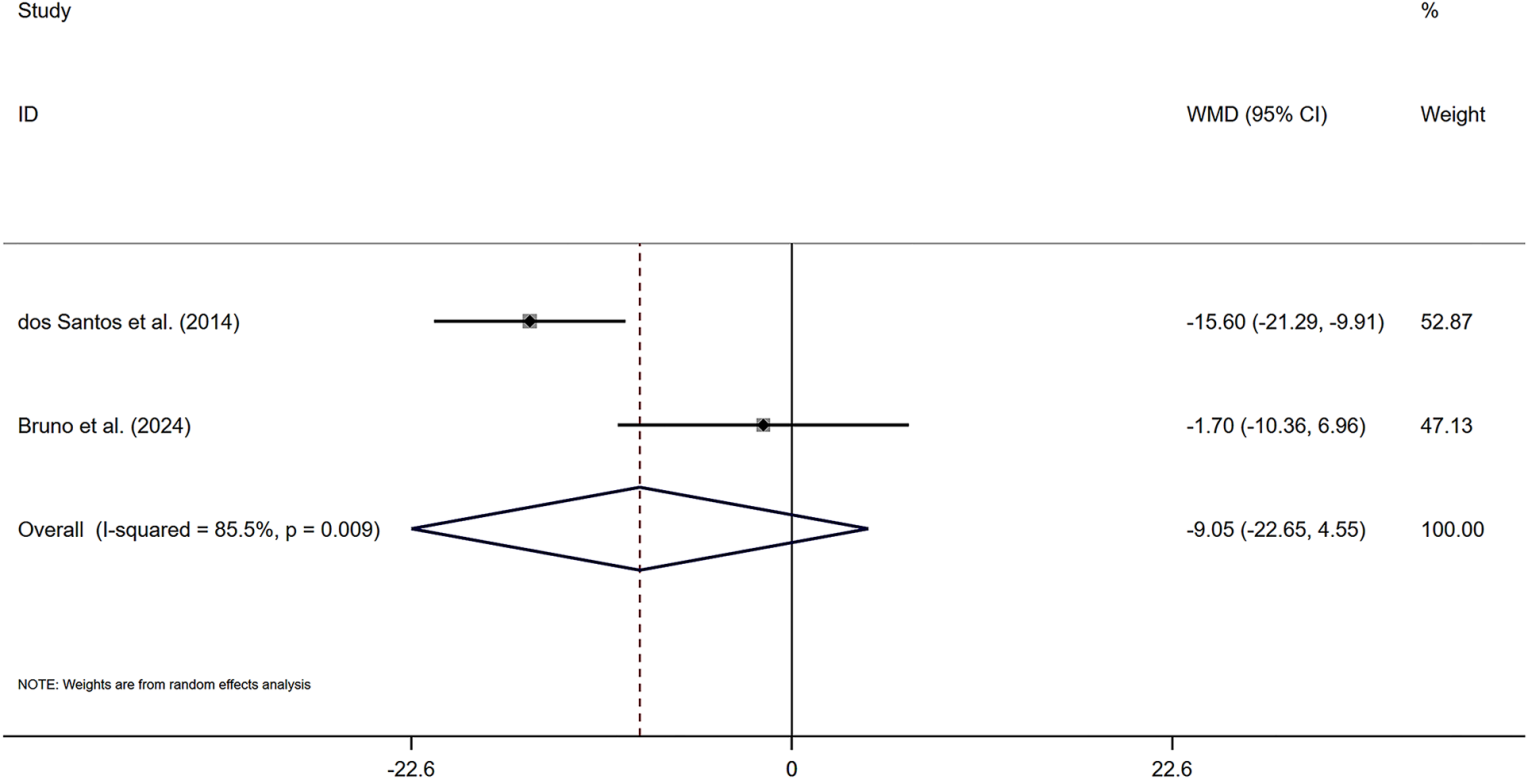
Forest plot of the effects of combined exercise on PP.

As shown in Figure 4, two included studies provided data on changes in pulse pressure among participants engaging in combined exercise, with a total of 33 individuals receiving the combined exercise intervention. The pooled statistical analysis did not yield a significant difference [*Z* = 1.3 (*P* > 0.05), 95% CI (−22.65, 4.55)], indicating that the effect of combined exercise on PP was not significant in the subjects. A sensitivity analysis (Supplementary Figure S3) confirmed the robustness of these findings.

### 3.5 Subgroup analysis

Subgroup analysis is a crucial method for exploring sources of heterogeneity in research to identify appropriate combined exercise programs. The results are presented in Table 4. The subgroup analysis results of SBP studies showed that in combined exercise programs, significant reductions in SBP were observed when the intensity of aerobic training (HRmax) was <85% (WMD = −11.6 mm Hg, *P* = 0.001), duration ≤12 weeks (WMD = −8.12 mm Hg, *P* = 0.001), weekly frequency ≥3 (WMD = −12.75 mm Hg, *P* = 0.001), weekly frequency <3 (WMD = −4.6 mm Hg, *P* = 0.011), total time <60 min (WMD = −10 mm Hg, *P* = 0.001), and the sequence was AE-RT (WMD = −9.14, *P* = 0.001). Additionally, regular combined exercise significantly improved SBP in individuals aged 60–70 (WMD = −12.59 mm Hg, *P* = 0.004), age >70 (WMD = −7.28 mm Hg, *P* = 0.015), those not taking antihypertensive medication (WMD = −6.34 mm Hg, *P* = 0.006), and those taking antihypertensive medication (WMD = −13.76 mm Hg, *P* = 0.002).

**Table 4:** Results of subgroup analysis.

Variable	Measurement	Subgroup	*N*	WMD	95% CI	*Z*	*P*-value	*I* ^2^
Aerobic training intensity	SBP	≥85% HRmax	2	−5.24	−10.86, 0.37	1.83	0.067	64.8%
		<85% HRmax	2	−11.6	−17.73, −5.46	3.71	0.001**	0%
	DBP	≥85% HRmax	2	−1.43	−3.22, 0.36	1.57	0.117	0%
		<85% HRmax	2	−5.25	−10.85, 0.34	1.84	0.066	0%
Duration	SBP	≤12 weeks	6	−8.12	−11.33, −4.91	4.95	0.001**	21.3%
		>12 weeks	3	−14.68	−32.55, 3.19	1.61	0.107	96.4%
	DBP	≤12 weeks	6	−4.62	−6.66, −2.58	4.44	0.001**	0%
		>12 weeks	3	−6.45	−14.19, 1.28	1.63	0.102	94.9%
Weekly frequency	SBP	≥3	7	−12.75	−20.45, −5.06	3.25	0.001**	89.3%
		<3	2	−4.6	−8.18, −1.04	2.53	0.011*	0%
	DBP	≥3	7	−6.42	−10.95, −1.9	2.78	0.005**	85.8%
		<3	2	−3.78	−6.25, −1.32	3	0.003**	0%
Total time	SBP	Time ≥ 60 min	2	−8.03	−17.2, 1.14	1.72	0.086	69.8%
		Time < 60 min	3	−10	−14.19, −5.81	4.68	0.001**	0%
	DBP	Time ≥ 60 min	2	−6.45	−13.56, 0.65	1.78	0.075	73.2%
		Time < 60 min	3	−4.8	−9.03, −0.57	2.22	0.026*	0%
Age	SBP	60–70	7	−12.59	−21.24, −3.94	2.85	0.004**	91%
		70+	2	−7.28	−13.14, −1.41	2.43	0.015*	41.4%
	DBP	60–70	7	−5.76	−10.67, −0.86	2.3	0.021*	87.3%
		70+	2	−5.79	−10.06, −1.53	2.66	0.008**	48.2%
Sequence	SBP	AE-RT	6	−9.14	−13.56, −4.73	4.06	0.001**	55.7%
		RT-AE	3	−13.26	−30.2, 3.68	1.53	0.125	95.7%
	DBP	AE-RT	6	−4.78	−8.05, −1.51	2.86	0.004**	51.9%
		RT-AE	3	−7.15	−13.4, −0.9	2.24	0.025*	89.1%
antihypertensive drugs	SBP	Not taking medication	4	−6.34	−10.84, −1.85	2.76	0.006**	58.7%
		Take medicine	5	−13.76	−22.51, −5.01	3.08	0.002**	85.1%
	DBP	Not taking medication	4	−4.24	−7.47, −1.01	2.57	0.01*	69.2%
		Take medicine	5	−7.32	−11.23, −3.41	3.67	0.001**	52.9%

Due to the small number of PP studies, no subgroup analysis was conducted for this study; *N*, number of studies.

* *P* < 0.05; **P < 0.01.

The subgroup analysis results of DBP studies indicated that in combined exercise programs, significant reductions in DBP were found when duration ≤12 weeks (WMD = −4.62 mm Hg, *P* = 0.001), weekly frequency ≥3 (WMD = −6.42 mm Hg, *P* = 0.005), weekly frequency <3 (WMD = −3.78 mm Hg, *P* = 0.003), total time <60 min (WMD = −4.8 mm Hg, *P* = 0.026), the sequence was AE-RT (WMD = −4.78 mm Hg, *P* = 0.004), and the sequence was RT-AE (WMD = −7.15 mm Hg, *P* = 0.025). Notably, regular combined exercise also significantly improved DBP in individuals aged 60–70 (WMD = −5.76 mm Hg, *P* = 0.021), age >70 (WMD = −5.79 mm Hg, *P* = 0.008), those taking antihypertensive medication (WMD = −7.32 mm Hg, *P* = 0.001), and those not taking antihypertensive medication (WMD = −4.24 mm Hg, *P* = 0.01).

## 4 Discussion

This meta-analysis has robustly demonstrated the substantial influence of a combined exercise regimen, integrating aerobic and resistance components, on blood pressure in elderly individuals with hypertension. Our findings indicate a pronounced decrease in both systolic and diastolic blood pressure, with an average reduction of 11.17 mm Hg and 5.93 mm Hg observed, respectively. The potential exists for enhanced blood pressure management through customized exercise plans tailored to the patient's needs. Notably, research indicates a 2.1% escalation in cardiovascular mortality risk for every 1 mmHg elevation in blood pressure (26).

This meta-analysis substantiates that a regimen intertwining aerobic with resistance exercise exerts a favorable influence on blood pressure reduction, thereby considerably diminishing cardiovascular mortality risk among the elderly. This is also important for elderly patients with high blood pressure. Elderly people over 60 years old were included in this analysis because hypertension has the highest incidence among elderly people, and patients with prehypertension or grade 1 hypertension have mild hypertension. By improving body function, disease treatment or prevention can be achieved, and there is a certain probability of gradually returning to normal. Scapinia et al. (27) conducted a meta-analysis that revealed a combined regimen of aerobic and resistance training to be more effective than aerobic exercise alone in managing blood pressure in patients with end-stage renal disease, exhibiting significantly greater efficacy. Loaiza-Betancur et al. (28) noted that while aerobic training alone did not result in significant reductions in blood pressure among elderly women, the combination of aerobic and resistance exercise led to substantial decreases. Furthermore, Laurent et al. (29) emphasized that the integration of aerobic and resistance exercises effectively reduces both systolic and diastolic blood pressure in middle-aged and older adults, irrespective of the order in which the exercises are executed. Our current meta-analysis further supports the significant hypotensive benefits of such combined exercise regimens across various populations, including hypertensive elderly individuals. Furthermore, in this meta-analysis, 211 subjects participated in combined exercise interventions, with only 11 subjects being excluded due to the effects of exercise. Combined exercise, characterized by low risk, moderate intensity, and high efficiency (30), is a worthwhile approach for anti-hypertensive intervention that merits promotion.

### 4.1 Influence of exercise intensity on blood pressure reduction in hypertensive patients

Subgroup analysis results indicate that within the combined exercise program, when the aerobic training intensity is HRmax < 85%, the intervention effect on SBP (WMD = −11.6 mm Hg, *P* = 0.001) is more pronounced. The benefits and risks associated with different exercise intensities for hypertensive patients are proportional, and Boutcher (31) et al. confirmed in their study that high-intensity interval training (>70% of maximal oxygen consumption, VO2max) can significantly reduce systolic blood pressure, diastolic blood pressure, and ambulatory blood pressure in patients with essential hypertension. Opinions among scholars regarding the effects of high-intensity aerobic exercise interventions vary. While the American Heart Association and the 2013 Hypertension Lifestyle Management Guidelines advocate for moderate-intensity aerobic exercise(50%–70% HRmax) for individuals with hypertension, they also support the inclusion of high-intensity aerobic exercise(80%–100% VO2max), provided the patients’ physical conditions allow it (32).Schneider et al. (33) demonstrated in their study that the greater the volume of resistance training, the better the blood pressure-lowering effect of combined exercise. In the combined exercise program, for every additional 5 sets of resistance training per week, SBP can be reduced by 0.5 mmHg. When the intensity of resistance training is at 50% or 80% of 1-RM, SBP can be reduced by 10 or 17 mmHg, and DBP can be reduced by 9 or 15 mmHg, respectively. However, some researchers have shown that high-intensity eccentric resistance exercise (maximal eccentric elbow flexor exercise) can impair endothelial function in young men, manifested as a decrease in endothelium-dependent vasodilation (reduced FMD) and an increase in central arterial stiffness (34). However, Aghaei Bahmanbeglou et al. (35) found in their study that high-intensity intermittent exercise (80%–100% VO2max) was not suitable for patients with severe cardiovascular diseases. Consequently, high-intensity exercise could serve as a favorable substitute for moderate-intensity workouts given its time efficiency and pronounced impact on blood pressure reduction. However, due to elevated exercise-associated risks, the application of high-intensity interventions should be cautiously implemented with participant safety as a paramount concern. Resistance training can improve muscle endurance and strength, so it is necessary to be careful in the use of resistance exercise.

Moderate- to low-intensity resistance exercises (30%–70% of 1-RM) have been shown to possess blood pressure-lowering effects and exert positive influences on arterial stiffness. In comparison to high-intensity resistance exercise, moderate-intensity exercise is deemed safer (36). Polito et al. (37) suggest that, to achieve blood pressure-lowering benefits in adults, the intensity of resistance exercise should be maintained between 60% and 80% of 1-RM. Therefore, regardless of whether aerobic exercise or resistance exercise is performed, each exercise intensity plays a unique role and holds inherent value. This is also an advantage of combined exercise, as each program can be more widely personalized according to the specific situation.

### 4.2 Association between the duration of exercise and blood pressure reduction

The results of the subgroup analysis revealed that combined exercise interventions lasting ≤12 weeks exhibited greater efficacy in reducing SBP (WMD = −8.12 mm Hg, *P* = 0.001) and DBP (WMD = −4.62 mm Hg, *P* = 0.001). Similarly, when the total exercise time was <60 min, it demonstrated a significant effect on lowering SBP (WMD = −10 mm Hg, *P* = 0.001) and DBP (WMD = −4.8 mm Hg, *P* = 0.026). These findings align with the minimum exercise duration recommended in the Eighth Joint National Committee Report (38) and by the AHA/ACC Lifestyle Work Group (39). This observation is further corroborated by the work of Xi et al. (40), who reported significant decreases in blood pressure among postmenopausal women following 12 weeks of combined aerobic and resistance training. These findings highlight the importance of this particular duration of exercise as a potential benchmark for effective intervention. Nonetheless, given the limited number of samples, it remains inconclusive whether ≤12 weeks or <60 min, of exercise can sustain long-term benefits; thus, future research with more randomized controlled trials is warranted to elucidate these effects.

### 4.3 The blood pressure-lowering mechanism of combined exercise

Exercise regulates blood pressure in hypertensive patients primarily through neural and humoral mechanisms. In neural regulation, exercise modifies the muscle sympathetic nervous activity (MSNA) and arterial baroreflex sensitivity (BRS) in hypertensive individuals. In humoral regulation, it involves vasoactive substances and the renin-angiotensin system. By acting on both humoral and neural systems, exercise triggers a cascade of molecular biological reactions related to cell proliferation and differentiation, leading to changes in arterial blood pressure (41), which constitutes the primary mechanism for blood pressure reduction through combined exercise.

The development of hypertension is closely linked to autonomic nervous dysfunction, with increased sympathetic nervous activity (SNA) recognized as a key player in its onset and progression. Aerobic exercise has the capacity to rectify dysfunctional sympathetic nervous systems. Masson et al. (42) demonstrated that aerobic exercise can delay hypertension development in rats by enhancing BRS. Besnier et al. (43) confirmed that aerobic exercise normalizes the overexcitation of MSNA, a neural regulatory mechanism that aids in reducing vascular smooth muscle tone. Unlike aerobic exercise, resistance exercise modulates the vagus nerve, inducing continuous changes in cardiac autonomic nerve function and sustained elevation of vagal tone in hypertensive patients, which is conducive to blood pressure control (44).

In humoral regulation, combined exercise promotes smooth muscle cell relaxation by alleviating inflammation and enhancing nitric oxide (NO) bioavailability, potentially playing a crucial role in preventing harmful vascular remodeling and fostering healthy blood pressure development (21, 45). Aerobic exercise has been shown to lower plasma endothelin-1 levels (46) and attenuate its vasoconstrictive effects in hypertensive patients, exerting a positive influence on vascular tone (47). In the context of insulin resistance mechanisms, the Nod-like receptor protein 3 (NLRP3) inflammasome plays a pivotal role. Both aerobic and resistance exercises can inhibit the excessive activation of inflammatory factors, including the NLRP3 inflammasome, thereby reducing inflammation and improving insulin sensitivity (48, 49). Blood lipids, as an important indicator of vascular health, are also closely related to hypertension. In studies on blood lipids, Fikenzer et al. (50) demonstrated that aerobic exercise can lower levels of total cholesterol (TC), triglycerides (TG), and low-density lipoprotein cholesterol (LDL-C). Wooten et al. (51) reported that resistance training over a 12-week period markedly reduced TC and LDL-C in postmenopausal women. Therefore, adopting a combined exercise approach may offer benefits for lipid metabolism and further influence blood pressure reduction. When considering adiposity, its accumulation has been associated with hypertension and dyslipidemia (52, 53). Willis et al. (54) found that resistance training alone was less effective than aerobic exercise in decreasing body fat; however, combined exercises over 12 weeks proved efficacious in lowering body fat percentage among hypertensive senior women (21). This suggests that ongoing combined exercise could be instrumental in maintaining lipid health and reducing cardiovascular disease (CVD) risk in hypertensive individuals by also promoting body fat reduction. Although extensive studies have investigated the individual mechanisms of blood pressure reduction by resistance and aerobic exercises, the combined effects of these modalities require further study. The link between combined exercise and arterial health has been shown, where arterial stiffness is exacerbated by the accrual of advanced glycation end products (AGEs) affecting the arterial wall's extracellular matrix (55–58). Pekas (58) found in their experiment that combined exercise reduced the brachial-ankle pulse wave velocity (baPWV) by 1 m/s in elderly women. This finding also holds clinical significance for elderly hypertensive populations, as previous studies have indicated that a 1.0 m/s increase in baPWV is associated with a 12% increase in cardiovascular disease risk (59). This suggests that combined exercise may have a role in delaying vascular remodeling. Furthermore, elderly individuals should be actively encouraged to enhance their muscle strength. The prevalence of sarcopenia is increasing among the elderly, and the decline in muscle strength can lead to insulin resistance, elevated inflammation levels, and endothelial dysfunction. Resistance training contributes to the development of muscle strength (60, 61).

### 4.4 Potential modulators of combined exercise

The regulatory influences of aerobic and resistance training on blood pressure among hypertensive patients have been firmly established in the literature. Empirical studies have demonstrated that combined exercise interventions achieve a more potent reduction in blood pressure than aerobic exercise administered in isolation (27, 62). This enhanced efficacy is presumably attributable to the incorporation of resistance training components within the combined exercise protocols. Resistance training encompasses numerous types, and different resistance exercises exhibit varied blood pressure-lowering effects in hypertensive patients, coupled with multifaceted health promotions for users. This offers a diverse array of options for elderly individuals to employ combined exercise as a therapeutic approach for hypertension.

In Henkin's meta-analysis (63), it was noted that resistance exercise led to a significant reduction in SBP by approximately 7 mm Hg and DBP by an average of 3 mm Hg among the elderly. Isometric resistance training (IRT), a prevalent form of resistance training, has been confirmed in a meta-analysis to effectively decrease SBP by about 7.47 mm Hg and DBP by approximately 3.17 mm Hg (64). Due to its cost-effectiveness, accessibility, scheduling flexibility, and ease of training, IRT has emerged as an ideal adjunct to combined exercise programs. A study by Olga Ribeiro-Torres et al. (65) reported that, compared to traditional resistance training, intermittent resistance training with rest intervals (IRRT) had a more favorable impact on hemodynamic responses in elderly patients with coronary artery disease, potentially offering advantages for cardiac rehabilitation. Individuals aged 45 and older, as well as those with hypertension, may derive greater benefits from IRT (66). However, it is crucial to acknowledge that reports suggest IRT may elevate the risk of mild cognitive impairment in postmenopausal women with a history of preeclampsia (67), necessitating careful consideration when selecting resistance training as a complementary hypertension therapy. Another study revealed that long-term dynamic resistance training (DRT) can reduce systolic and diastolic blood pressure in adults by an average of approximately 2.6 mm Hg and 3.1 mm Hg, respectively (68). Furthermore, DRT provides additional benefits such as increased muscle mass, strength, and function (69).

Contemporary clinical investigations have indicated that blood flow restriction (BFR) training can beneficially impact blood pressure management in senior populations. Zhang et al. (70) propose that low-load blood flow restriction training (LL-BFR) offers benefits for elderly individuals, leading to an average decrease of around −6.59 mm Hg in SBP. Furthermore, LL-BFR training can significantly improve the flow-mediated dilation (FMD) function in the elderly, with an average increase of about 1.3%. An enhanced FMD benefits cardiovascular health and helps to improve and prevent arteriosclerosis. Enhancements in FMD are intrinsically linked with improvements in endothelial function. Hypoxia-stimulated enhancement of vascular endothelial growth factor (VEGF) activity is considered a crucial process by which training involving BFR engenders enhancement in FMD (71). During low-load blood flow restriction (LL-BFR) training sessions, localized ischemic reperfusion may induce the secretion of vascular endothelial growth factor (VEGF) from vascular endothelial and skeletal muscle cells, and concurrently provoke a surge in human growth hormone (HGH) levels (72). Both processes are known to augment nitric oxide synthase (NOS) expression (73), and alongside the cardioprotective outcomes of ischemic preconditioning (74), they enhance nitric oxide bioavailability, fortifying endothelial function (75, 76). This chain of events has a positive ripple effect on the overall well-being of individuals with hypertension. A blend of aerobic and resistance exercises emerges as a promising avenue for enhancing the health of elderly individuals with hypertension.

Considering the notably elevated incidence of hypertension among the elderly population, our research endeavors to assess the impact of integrating aerobic and resistance exercises specifically on blood pressure in this demographic. Furthermore, we strive to elucidate the mechanisms underlying the effects of this combined exercise regimen on blood pressure and to identify key regulatory factors involved. What sets our research apart from previous studies, which primarily focused on either aerobic or resistance exercise in isolation, is our incorporation of both exercise modalities. It is worth noting that this combined approach was well-received by most participants due to its shorter duration and reduced intensity. By conducting a meta-analysis, we provide valuable insights into the antihypertensive effects of this exercise regimen in elderly patients with hypertension, which holds significant implications for their management and treatment.

## 5 Limitations

This study has several limitations. Firstly, the relatively scarce number and sample size of studies on the chronic effects of combined aerobic and resistance exercise in elderly patients with prehypertension or stage 1 hypertension in the databases have imposed constraints, directly affecting the final results of the meta-analysis. Secondly, the meta-analysis results exhibited high heterogeneity, which is unavoidable due to the variations in specific exercise programs, intensities, and intervention durations among each study. The small scale and limited data hindered the exploration of heterogeneity sources when conducting subgroup analyses. Thirdly, due to the limited evidence, some parts of the discussion may be speculative, and future studies are needed to employ objective indicators for validation.

## 6 Conclusion

This meta-analysis confirms the effectiveness of integrating aerobic and resistance exercises for managing hypertension in older adults, notably reducing blood pressure and lowering the risk of cardiovascular issues. It emphasizes that exercise programs of 12 weeks or shorter are especially beneficial in lowering blood pressure for the elderly, underscoring the critical role of intervention duration. The positive impact of these combined exercise modalities on hypertension is believed to stem from a variety of mechanisms. Together, these activities improve several physiological functions essential for blood pressure regulation, showcasing the holistic benefits of exercise in treating hypertension. Nonetheless, the study's findings are moderated by certain limitations, such as the small sample sizes and the narrow scope of studies reviewed. The observed Heterogeneity and limitations highlight an urgent need for additional rigorous randomized controlled trials. Such research is essential to verify the advantages of customized exercise regimens that incorporate both aerobic and resistance elements for aging individuals with hypertension. Further studies are also needed to elucidate the specific pathways through which these exercises achieve blood pressure reduction, providing deeper understanding of their therapeutic potential.

## Data Availability

The original contributions presented in the study are included in the article/Supplementary Material, further inquiries can be directed to the corresponding author.

## References

[1] Zhou B Perel P Mensah GA Ezzati M. Global epidemiology, health burden and effective interventions for elevated blood pressure and hypertension. Nat Rev Cardiol. (2021) 18(11):785–802. 10.1038/s41569-021-00559-8 34050340 PMC8162166

[2] Al-Makki A DiPette D Whelton PK Murad MH Mustafa RA Acharya S Hypertension pharmacological treatment in adults: a world health organization guideline executive summary. Hypertension. (2022) 79(1):293–301. 10.1161/HYPERTENSIONAHA.121.18192 34775787 PMC8654104

[3] Benetos A Petrovic M Strandberg T. Hypertension management in older and frail older patients. Circ Res. (2019) 124(7):1045–60. 10.1161/CIRCRESAHA.118.313236 30920928

[4] Roh J Rhee J Chaudhari V Rosenzweig A. The role of exercise in cardiac aging: from physiology to molecular mechanisms. Circ Res. (2016) 118(2):279–95. 10.1161/CIRCRESAHA.115.305250 26838314 PMC4914047

[5] Chobanian AV Bakris GL Black HR Cushman WC Green LA Izzo JL Jr The seventh report of the joint national committee on prevention, detection, evaluation, and treatment of high blood pressure: the JNC 7 report. JAMA. (2003) 289(19):2560–72. 10.1001/jama.289.19.2560 12748199

[6] Olsen MH Angell SY Asma S Boutouyrie P Burger D Chirinos JA A call to action and a lifecourse strategy to address the global burden of raised blood pressure on current and future generations: the lancet commission on hypertension. Lancet. (2016) 388(10060):2665–712. 10.1016/S0140-6736(16)31134-5 27671667

[7] Esmailiyan M Amerizadeh A Vahdat S Ghodsi M Doewes RI Sundram Y. Effect of different types of aerobic exercise on individuals with and without hypertension: an updated systematic review. Curr Probl Cardiol. (2023) 48(3):101034. 10.1016/j.cpcardiol.2021.101034 34718034

[8] Halliwill JR Buck TM Lacewell AN Romero SA. Postexercise hypotension and sustained postexercise vasodilatation: what happens after we exercise? Exp Physiol. (2013) 98(1):7–18. 10.1113/expphysiol.2011.058065 22872658

[9] Luttrell MJ Halliwill JR. Recovery from exercise: vulnerable state, window of opportunity, or crystal ball? Front Physiol. (2015) 6:204. 10.3389/fphys.2015.00204 26257656 PMC4510411

[10] Cornelissen VA Smart NA. Exercise training for blood pressure: a systematic review and meta-analysis. J Am Heart Assoc. (2013) 2(1):e004473. 10.1161/JAHA.112.004473 23525435 PMC3603230

[11] Hashida R Kawaguchi T Bekki M. Aerobic versus resistance exercise in non-alcoholic fatty liver disease: a systematic review. J Hepatol. (2017) 66(1):142–52. 10.1016/j.jhep.2016.08.023 27639843

[12] Taylor AC McCartney N Kamath MV Wiley RL. Isometric training lowers resting blood pressure and modulates autonomic control. Med Sci Sports Exerc. (2003) 35(2):251–6. 10.1249/01.MSS.0000048725.15026.B5 12569213

[13] Barcellos FC Del Vecchio FB Reges A Mielke G Santos IS Umpierre D Exercise in patients with hypertension and chronic kidney disease: a randomized controlled trial. J Hum Hypertens. (2018) 32(6):397–407. 10.1038/s41371-018-0055-0 29615792

[14] Fragala MS Cadore EL Dorgo S Izquierdo M Kraemer WJ Peterson MD Resistance training for older adults: position statement from the national strength and conditioning association. J Strength Cond Res. (2019) 33(8):2019–52. 10.1519/JSC.0000000000003230 31343601

[15] Hayashino Y Jackson JL Fukumori N Nakamura F Fukuhara S. Effects of supervised exercise on lipid profiles and blood pressure control in people with type 2 diabetes mellitus: a meta-analysis of randomized controlled trials. Diabetes Res Clin Pract. (2012) 98(3):349–60. 10.1016/j.diabres.2012.10.004 23116535

[16] Smart NA Waldron M Ismail H Giallauria F Vigorito C Cornelissen V Validation of a new tool for the assessment of study quality and reporting in exercise training studies: TESTEX. Int J Evid Based Healthc. (2015) 13(1):9–18. 10.1097/XEB.0000000000000020 25734864

[17] Bavaresco Gambassi B Chaves LFC Sousa TMDS Ribeiro MJS Souza TA Schwingel PA. Short-duration dynamic power training with elastic bands combined with endurance training: a promising approach to hypertension management in older adults. J Hypertens. (2024) 42(4):735–42. 10.1097/HJH.0000000000003681 38441186

[18] Miura H Takahashi Y Maki Y Sugino M. Effects of exercise training on arterial stiffness in older hypertensive females. Eur J Appl Physiol. (2015) 115(9):1847–54. 10.1007/s00421-015-3168-y 25869875

[19] Sousa N Mendes R Abrantes C Sampaio J Oliveira J. A randomized 9-month study of blood pressure and body fat responses to aerobic training versus combined aerobic and resistance training in older men. Exp Gerontol. (2013) 48(8):727–33. 10.1016/j.exger.2013.04.008 23628502

[20] Dos Santos ES Asano RY Filho IG Lopes NL Panelli P da C Nascimento D Acute and chronic cardiovascular response to 16 weeks of combined eccentric or traditional resistance and aerobic training in elderly hypertensive women: a randomized controlled trial. J Strength Cond Res. (2014) 28(11):3073–84. 10.1519/JSC.0000000000000537 24845208

[21] Son WM Sung KD Cho JM Park SY. Combined exercise reduces arterial stiffness, blood pressure, and blood markers for cardiovascular risk in postmenopausal women with hypertension. Menopause. (2017) 24(3):262–8. 10.1097/GME.0000000000000765 27779565

[22] Ruangthai R Phoemsapthawee J. Combined exercise training improves blood pressure and antioxidant capacity in elderly individuals with hypertension. J Exerc Sci Fitness. (2019) 17(2):67–76. 10.1016/j.jesf.2019.03.001 PMC6430041 30949214

[23] Ruangthai R Phoemsapthawee J Makaje N Phimphaphorn P. Comparative effects of water- and land-based combined exercise training in hypertensive older adults. Arch Gerontol Geriatr. (2020) 90:104164. 10.1016/j.archger.2020.104164 32650155

[24] Barone BB Wang NY Bacher AC Stewart KJ. Decreased exercise blood pressure in older adults after exercise training: contributions of increased fitness and decreased fatness. Br J Sports Med. (2009) 43(1):52–6. 10.1136/bjsm.2008.050906 18728054 PMC3725468

[25] de Oliveira SN Pereira Moro AR Polito MD Helena de Jesus J de Souza Bezerra E. Effects of concurrent training with elastic tubes in hypertensive patients: a blind controlled randomized clinical trial. Exp Aging Res. (2020) 46(1):68–82. 10.1080/0361073X.2019.1693030 31736406

[26] Li Y Thijs L Hansen TW Kikuya M Boggia J Richart T Prognostic value of the morning blood pressure surge in 5645 subjects from 8 populations. Hypertension. (2010) 55(4):1040–8. 10.1161/HYPERTENSIONAHA.109.137273 20212273

[27] Scapini KB Bohlke M Moraes OA Rodrigues CG Inácio JF Sbruzzi G Combined training is the most effective training modality to improve aerobic capacity and blood pressure control in people requiring haemodialysis for end-stage renal disease: systematic review and network meta-analysis. J Physiother. (2019) 65(1):4–15. 10.1016/j.jphys.2018.11.008 30581137

[28] Loaiza-Betancur AF Chulvi-Medrano I Díaz-López VA Gómez-Tomás C. The effect of exercise training on blood pressure in menopause and postmenopausal women: a systematic review of randomized controlled trials. Maturitas. (2021) 149:40–55. 10.1016/j.maturitas.2021.05.005 34108092

[29] Laurent S Cockcroft J Van Bortel L Boutouyrie P Giannattasio C Hayoz D Expert consensus document on arterial stiffness: methodological issues and clinical applications. Eur Heart J. (2006) 27(21):2588–605. 10.1093/eurheartj/ehl254 17000623

[30] Corso LM Macdonald HV Johnson BT Farinatti P Livingston J Zaleski AL Is concurrent training efficacious antihypertensive therapy? A meta-analysis. Med Sci Sports Exercise. (2016) 48(12):2398–406. 10.1249/MSS.0000000000001056 27471784

[31] Boutcher YN Boutcher SH. Exercise intensity and hypertension: what’s new? J Hum Hypertens. (2017) 31(3):157–64. 10.1038/jhh.2016.62 27604656

[32] Virani SS Alonso A Aparicio HJ Benjamin EJ Bittencourt MS Callaway CW Heart disease and stroke statistics-2021 update: a report from the American Heart Association. Circulation. (2021) 143(8):e254–743. 10.1161/CIR.0000000000000950 33501848 PMC13036842

[33] Schneider VM Domingues LB Umpierre D Tanaka H Ferrari R. Exercise characteristics and blood pressure reduction after combined aerobic and resistance training: a systematic review with meta-analysis and meta-regression. J Hypertens. (2023) 41(7):1068–76. 10.1097/HJH.0000000000003455 37115856

[34] Choi Y Akazawa N Zempo-Miyaki A Ra SG Shiraki H Ajisaka R Acute effect of high-intensity eccentric exercise on vascular endothelial function in young men. J Strength Cond Res. (2016) 30(8):2279–85. 10.1519/JSC.0000000000000536 24832967

[35] Aghaei Bahmanbeglou N Ebrahim K Maleki M Nikpajouh A Ahmadizad S. Short-duration high-intensity interval exercise training is more effective than long duration for blood pressure and arterial stiffness but not for inflammatory markers and lipid profiles in patients with stage 1 hypertension. J Cardiopulm Rehabil Prev. (2019) 39(1):50–5. 10.1097/HCR.0000000000000377 30586113

[36] Zhang Y Zhang YJ Ye W Korivi M. Low-to-Moderate-Intensity resistance exercise effectively improves arterial stiffness in adults: evidence from systematic review, meta-analysis, and meta-regression analysis. Front Cardiovasc Med. (2021) 8:738489. Published 2021 October 11. 10.3389/fcvm.2021.738489 34708090 PMC8544752

[37] Polito MD Dias JR Jr Papst RR. Resistance training to reduce resting blood pressure and increase muscle strength in users and non-users of anti-hypertensive medication: a meta-analysis. Clin Exp Hypertens. (2021) 43(5):474–85. 10.1080/10641963.2021.1901111 33784899

[38] James PA Oparil S Carter BL Cushman WC Dennison-Himmelfarb C Handler J 2014 evidence-based guideline for the management of high blood pressure in adults: report from the panel members appointed to the Eighth Joint National Committee (JNC 8). JAMA. (2014) 311(5):507–20. 10.1001/jama.2013.284427 24352797

[39] Eckel RH Jakicic JM Ard JD de Jesus JM Houston Miller N Hubbard VS 2013 AHA/ACC guideline on lifestyle management to reduce cardiovascular risk: a report of the American College of Cardiology/American Heart Association task force on practice guidelines. Circulation. (2014) 129(25 Suppl 2):S76–99. 10.1161/01.cir.0000437740.48606.d1 Epub 2013 November 12. Erratum in: Circulation. 2014 Jun 24;129(25 Suppl 2):S100-1. Erratum in: Circulation. 2015 January 27;131(4):e326. PMID: 24222015. 24222015

[40] Xi H He Y Niu Y Sui X Zhang J Zhu R Effect of combined aerobic and resistance exercise on blood pressure in postmenopausal women: a systematic review and meta-analysis of randomized controlled trials. Exp Gerontol. (2021) 155:111560. 10.1016/j.exger.2021.111560 34560198

[41] Bernardo BC McMullen JR. Molecular aspects of exercise-induced cardiac remodeling. Cardiol Clin. (2016) 34(4):515–30. 10.1016/j.ccl.2016.06.002 27692221

[42] Masson GS Costa TS Yshii L Fernandes DC Soares PP Laurindo FR Time-dependent effects of training on cardiovascular control in spontaneously hypertensive rats: role for brain oxidative stress and inflammation and baroreflex sensitivity. PloS one. (2014) 9(5):e94927. 10.1371/journal.pone.0094927 24788542 PMC4006803

[43] Besnier F Labrunée M Pathak A Pavy-Le Traon A Galès C Sénard JM Exercise training-induced modification in autonomic nervous system: an update for cardiac patients. Ann Phys Rehabil Med. (2017) 60(1):27–35. 10.1016/j.rehab.2016.07.002 27542313

[44] Taylor KA Wiles JD Coleman DD Sharma R O’driscoll JM. Continuous cardiac autonomic and hemodynamic responses to isometric exercise. Med Sci Sports Exercise. (2017) 49(8):1511–9. 10.1249/MSS.0000000000001271 28708775

[45] Bellien J Favre J Iacob M Gao J Thuillez C Richard V Arterial stiffness is regulated by nitric oxide and endothelium-derived hyperpolarizing factor during changes in blood flow in humans. Hypertension. (2010) 55(3):674–80. 10.1161/HYPERTENSIONAHA.109.142190 20083732

[46] Nyberg M Mortensen SP Hellsten Y. Physical activity opposes the age-related increase in skeletal muscle and plasma endothelin-1 levels and normalizes plasma endothelin-1 levels in individuals with essential hypertension. Acta Physiol. (2013) 207(3):524–35. 10.1111/apha.12048 23227981

[47] Dow CA Stauffer BL Brunjes DL Greiner JJ DeSouza CA. Regular aerobic exercise reduces endothelin-1-mediated vasoconstrictor tone in overweight and obese adults. Exp Physiol. (2017) 102(9):1133–42. 10.1113/EP086454 28635124

[48] Liang F Huang T Li B Zhao Y Zhang X Xu B. High-intensity interval training and moderate-intensity continuous training alleviate β-amyloid deposition by inhibiting NLRP3 inflammasome activation in APPswe/PS1dE9 mice. Neuroreport. (2020) 31(5):425–32. 10.1097/WNR.0000000000001429 32150150

[49] Mardare C Krüger K Liebisch G Seimetz M Couturier A Ringseis R Endurance and resistance training affect high fat diet-induced increase of ceramides, inflammasome expression, and systemic inflammation in mice. J Diabetes Res. (2016) 2016:4536470. 10.1155/2016/4536470 26788518 PMC4691630

[50] Fikenzer K Fikenzer S Laufs U Werner C. Effects of endurance training on serum lipids. Vasc Pharmacol. (2018) 101:9–20. 10.1016/j.vph.2017.11.005 29203287

[51] Wooten JS Phillips MD Mitchell JB Patrizi R Pleasant RN Hein RM Resistance exercise and lipoproteins in postmenopausal women. Int J Sports Med. (2011) 32(1):7–13. 10.1055/s-0030-1268008 21086242 PMC3354704

[52] Jiang SZ Lu W Zong XF Ruan HY Liu Y. Obesity and hypertension. Exp Ther Med. (2016) 12(4):2395–9. 10.3892/etm.2016.3667 27703502 PMC5038894

[53] Ito H Nakasuga K Ohshima A Sakai Y Maruyama T Kaji Y Excess accumulation of body fat is related to dyslipidemia in normal-weight subjects. Int J Obes Relat Metab Disord. (2004) 28(2):242–7. 10.1038/sj.ijo.0802528 14610531

[54] Willis LH Slentz CA Bateman LA Shields AT Piner LW Bales CW Effects of aerobic and/or resistance training on body mass and fat mass in overweight or obese adults. J Appl Physiol. (2012) 113(12):1831–7. 10.1152/japplphysiol.01370.2011 23019316 PMC3544497

[55] Costa D Barbalho MC Miguel GP Forti EM Azevedo JL. The impact of obesity on pulmonary function in adult women. Clinics (Sao Paulo). (2008) 63(6):719–24. 10.1590/S1807-59322008000600002 19060990 PMC2664268

[56] Lakatta EG Levy D. Arterial and cardiac aging: major shareholders in cardiovascular disease enterprises: part I: aging arteries: a “set up” for vascular disease. Circulation. (2003) 107(1):139–46. 10.1161/01.CIR.0000048892.83521.58 12515756

[57] Zieman SJ Melenovsky V Kass DA. Mechanisms, pathophysiology, and therapy of arterial stiffness. Arterioscler Thromb Vasc Biol. (2005) 25(5):932–43. 10.1161/01.ATV.0000160548.78317.29 15731494

[58] Pekas EJ Shin J Son WM Headid RJ 3rd Park SY. Habitual combined exercise protects against age-associated decline in vascular function and lipid profiles in elderly postmenopausal women. Int J Environ Res Public Health. (2020) 17(11):3893. 10.3390/ijerph17113893 32486335 PMC7312892

[59] Vlachopoulos C Aznaouridis K Terentes-Printzios D Ioakeimidis N Stefanadis C. Prediction of cardiovascular events and all-cause mortality with brachial-ankle elasticity index: a systematic review and meta-analysis. Hypertension. (2012) 60(2):556–62. 10.1161/HYPERTENSIONAHA.112.194779 22733468

[60] Maslow AL Sui X Colabianchi N Hussey J Blair SN. Muscular strength and incident hypertension in normotensive and prehypertensive men. Med Sci Sports Exerc. (2010) 42(2):288–95. 10.1249/MSS.0b013e3181b2f0a4 19927030 PMC2809142

[61] Consitt LA Dudley C Saxena G. Impact of endurance and resistance training on skeletal muscle glucose metabolism in older adults. Nutrients. (2019) 11(11):2636. 10.3390/nu11112636 31684154 PMC6893763

[62] Mannucci E Bonifazi A Monami M. Comparison between different types of exercise training in patients with type 2 diabetes mellitus: a systematic review and network metanalysis of randomized controlled trials. Nutr Metab Cardiovasc Dis. (2021) 31(7):1985–92. 10.1016/j.numecd.2021.02.030 33965297

[63] Henkin JS Pinto RS Machado CLF Wilhelm EN. Chronic effect of resistance training on blood pressure in older adults with prehypertension and hypertension: a systematic review and meta-analysis. Exp Gerontol. (2023) 177:112193. 10.1016/j.exger.2023.112193 37121334

[64] Baffour-Awuah B Pearson MJ Dieberg G Smart NA. Isometric resistance training to manage hypertension: systematic review and meta-analysis. Curr Hypertens Rep. (2023) 25(4):35–49. 10.1007/s11906-023-01232-w 36853479 PMC10014822

[65] Ribeiro-Torres O de Sousa AFM Iglesias-Soler E Fontes-Villalba M Zouhal H Carré F Lower cardiovascular stress during resistance training performed with inter-repetition rests in elderly coronary patients. Medicina (Kaunas). (2020) 56(6):264. 10.3390/medicina56060264 32481634 PMC7353896

[66] Inder JD Carlson DJ Dieberg G McFarlane JR Hess NC Smart NA. Isometric exercise training for blood pressure management: a systematic review and meta-analysis to optimize benefit. Hypertens Res. (2016) 39(2):88–94. 10.1038/hr.2015.111 26467494

[67] Miller KB Miller VM Harvey RE Ranadive SM Joyner MJ Barnes JN. Augmented cerebral blood velocity in response to isometric handgrip exercise in women with a history of preeclampsia. Am J Physiology Regul Integr Comp Physiol. (2019) 317(6):R834–9. 10.1152/ajpregu.00280.2019 PMC6962623 31663771

[68] Cornelissen VA Fagard RH Coeckelberghs E Vanhees L. Impact of resistance training on blood pressure and other cardiovascular risk factors: a meta-analysis of randomized, controlled trials. Hypertension. (2011) 58(5):950–8. 10.1161/HYPERTENSIONAHA.111.177071 21896934

[69] Abrahin O Moraes-Ferreira R Cortinhas-Alves EA Guerreiro JF. Is resistance training alone an antihypertensive therapy? A meta-analysis. J Hum Hypertens. (2021) 35(9):769–75. 10.1038/s41371-021-00582-9 34321596

[70] Zhang T Tian G Wang X. Effects of low-load blood flow restriction training on hemodynamic responses and vascular function in older adults: a meta-analysis. Int J Environ Res Public Health. (2022) 19(11):6750. 10.3390/ijerph19116750 35682336 PMC9180641

[71] Tammela T Enholm B Alitalo K Paavonen K. The biology of vascular endothelial growth factors. Cardiovasc Res. (2005) 65(3):550–63. 10.1016/j.cardiores.2004.12.002 15664381

[72] Ji JW Mac Gabhann F Popel AS. Skeletal muscle VEGF gradients in peripheral arterial disease: simulations of rest and exercise. Am J Physiol Heart Circ Physiol. (2007) 293(6):H3740–9. 10.1152/ajpheart.00009.2007 17890434

[73] Uematsu M Ohara Y Navas JP Nishida K Murphy TJ Alexander RW Regulation of endothelial cell nitric oxide synthase mRNA expression by shear stress. Am J Physiol. (1995) 269(6 Pt 1):C1371–8. 10.1152/ajpcell.1995.269.6.C1371 8572165

[74] Bailey TG Birk GK Cable NT Atkinson G Green DJ Jones H Remote ischemic preconditioning prevents reduction in brachial artery flow-mediated dilation after strenuous exercise. Am J Physiol Heart Circ Physiol. (2012) 303(5):H533–8. 10.1152/ajpheart.00272.2012 22730390

[75] Strijdom H Friedrich SO Hattingh S Chamane N Lochner A. Hypoxia-induced regulation of nitric oxide synthase in cardiac endothelial cells and myocytes and the role of the PI3-K/PKB pathway. Mol Cell Biochem. (2009) 321(1-2):23–35. 10.1007/s11010-008-9906-2 18791856

[76] Niebauer J Cooke JP. Cardiovascular effects of exercise: role of endothelial shear stress. J Am Coll Cardiol. (1996) 28(7):1652–60. 10.1016/S0735-1097(96)00393-2 8962548

