# Supplementary material for: Physiological characteristics of blood pressure responses after combined exercise in elderly hypertensive patients: a systematic review and meta-analysis
Source: Front Cardiovasc Med. 2024 Oct 25;11:1404127. doi: 10.3389/fcvm.2024.1404127 (PMC11543474; doi:10.3389/fcvm.2024.1404127)
Supplement: Supplementary file 1 [file Datasheet1.pdf]

Supplementary Material

Zhiheng Li[1], Moran LV[1], Zhen Li[1], Wei Gao[1], Ming Li[1*]

*** Correspondence:**
Corresponding Author
baomei005@163.com

1 Supplementary Data

A search method for retrieving documents on Pubmed

((((((((((((((((((("Exercise"[Mesh])) OR (Exercises[Title/Abstract])) OR (Physical Activity[Title/Abstract])) OR (Activities, Physical[Title/Abstract])) OR (Activity, Physical[Title/Abstract])) OR (Physical Activities[Title/Abstract])) OR (Exercise, Physical[Title/Abstract])) OR (Exercises, Physical[Title/Abstract])) OR (Physical Exercise[Title/Abstract])) OR (Physical Exercises[Title/Abstract])) OR (Acute Exercise[Title/Abstract])) OR (Isometric Exercises[Title/Abstract])) OR (Aerobic Exercise[Title/Abstract])) OR (Aerobic Exercises[Title/Abstract])) OR (Exercises, Aerobic[Title/Abstract])) OR ((((("High-Intensity Interval Training"[Mesh]) OR (High Intensity Interval Training[Title/Abstract])) OR (High-Intensity Interval Trainings[Title/Abstract])) OR (Interval Training, High-Intensity[Title/Abstract])) OR (Interval Trainings, High-Intensity[Title/Abstract]))) AND ((((((((((((("Resistance Training"[Mesh]) OR (Training, Resistance[Title/Abstract])) OR (Strength Training[Title/Abstract])) OR (Training, Strength[Title/Abstract])) OR (Weight-Lifting Strengthening Program[Title/Abstract])) OR (Strengthening Program, Weight-Lifting[Title/Abstract])) OR (Strengthening Programs, Weight-Lifting[Title/Abstract])) OR (Weight Lifting Strengthening Program[Title/Abstract])) OR (Weight-Lifting Strengthening Programs[Title/Abstract])) OR (Weight-Lifting Exercise Program[Title/Abstract])) OR (Exercise Program, Weight-Lifting[Title/Abstract])) OR (Exercise Programs, Weight-Lifting[Title/Abstract])) OR (Weight Lifting Exercise Program[Title/Abstract]))) OR (combined exercise[Title/Abstract])) OR (Concurrent training[Title/Abstract])) AND (((((("Blood Pressure"[Mesh]) OR (Pressure, Blood[Title/Abstract])) OR (Diastolic Pressure[Title/Abstract])) OR (Pressure, Diastolic[Title/Abstract])) OR (Pulse Pressure[Title/Abstract])) OR (Pressure, Pulse[Title/Abstract]))) AND (((("Randomized Controlled Trial" [Publication Type]) OR (Randomized Controlled Trial[Title/Abstract])) OR (randomized[Title/Abstract])) OR (placebo[Title/Abstract]))

2 Supplementary Figures

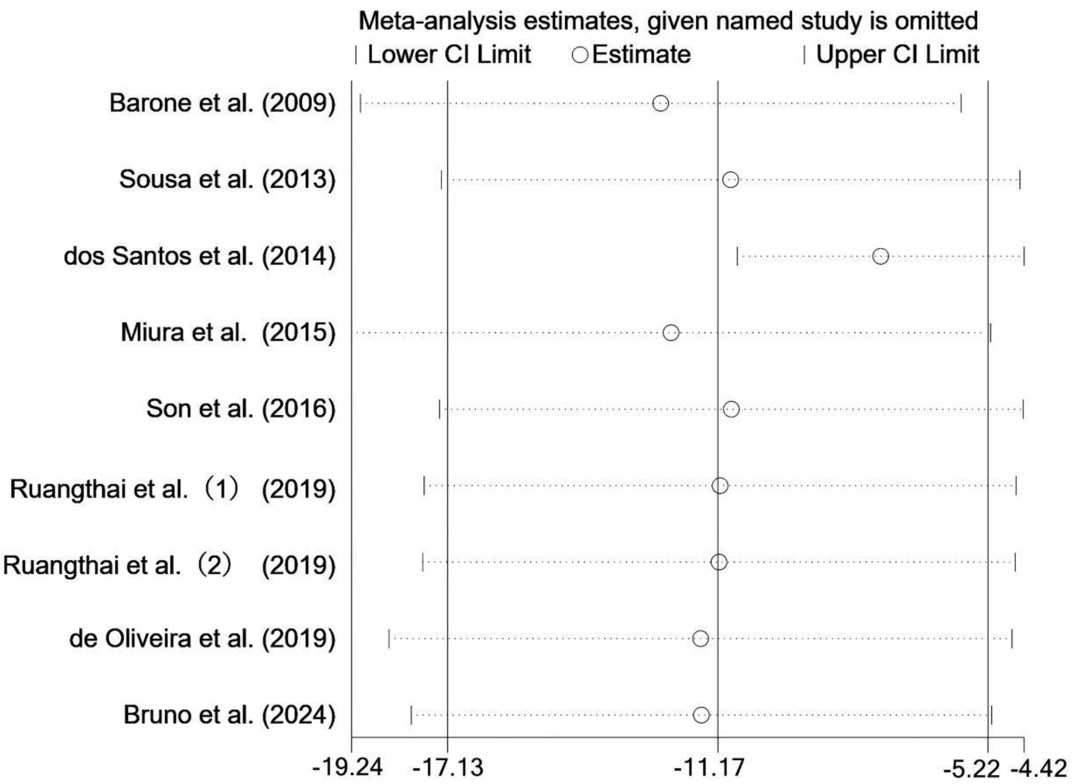

Supplementary Figure 1. Sensitivity analysis of the effects of combined exercise on subjects' SBP

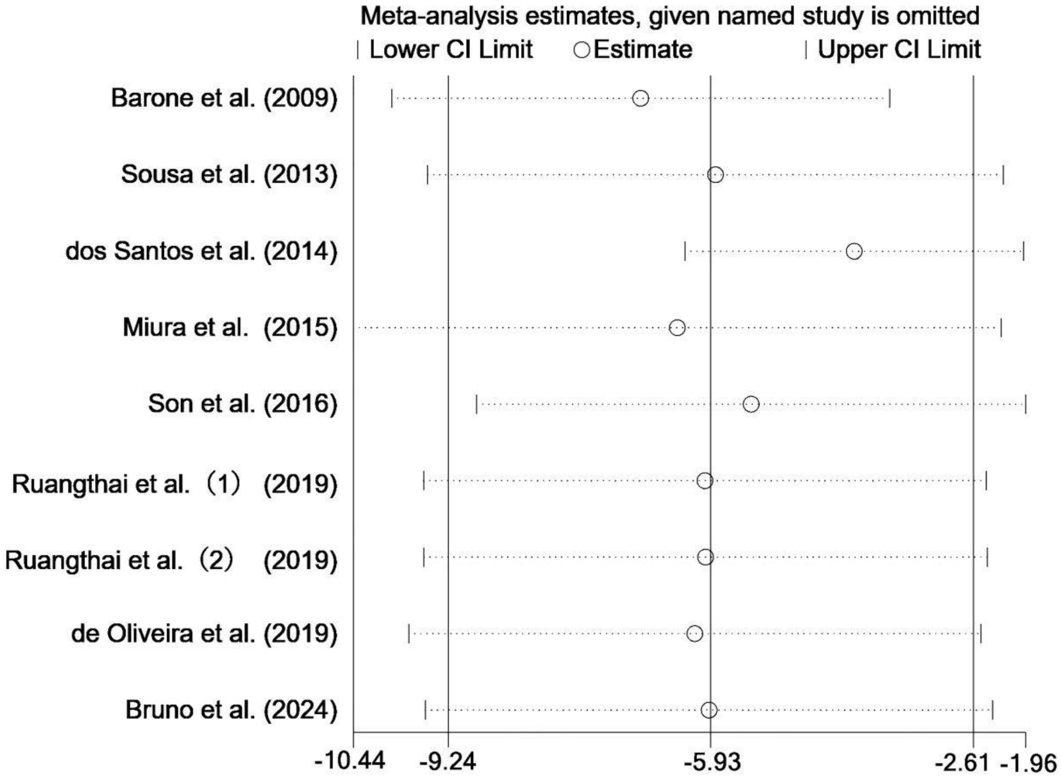

Supplementary Figure 2. Sensitivity analysis of the effects of combined exercise on DBP

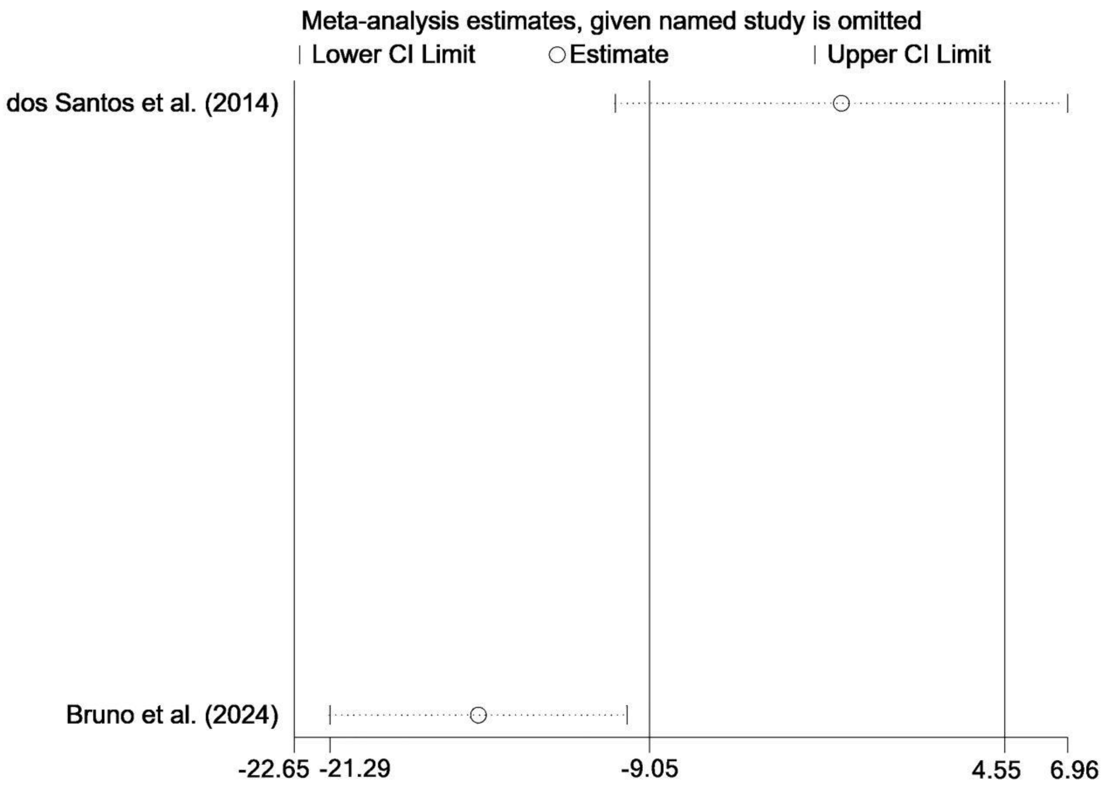

Supplementary Figure 3. Sensitivity analysis of the effects of combined exercise on PP

Data availability and sources

The authors confirm that the study was conducted without any commercial or financial connections. All the data on which the results are based can be found in publicly available databases.

Abbreviations

SBP: systolic Blood pressure

DBP: diastolic Blood pressure

RCTs: Randomized Controlled Trials

NO: Nitric oxide

baPWV: Brachial-ankle Pulse Wave Velocity

IRT: Isometric Resistance Training

BFR: Blood Flow Restriction Training

FMD: Flow-mediated dilation

VEGF: Vascular endothelial growth factor

FUNDING

General Project of Fujian Social Science Foundation (FJ2024B092)

Conflict of Interest

The authors affirm that the study was conducted without any affiliations with commercial entities or financial interests that could present a potential conflict of interest.
